# Binding and Inhibition of Spermidine Synthase from *Plasmodium falciparum* and Implications for *In Vitro* Inhibitor Testing

**DOI:** 10.1371/journal.pone.0163442

**Published:** 2016-09-23

**Authors:** Janina Sprenger, Jannette Carey, Bo Svensson, Verena Wengel, Lo Persson

**Affiliations:** 1 Center for Molecular Protein Science, Lund University, SE-221 00, Lund, Sweden; 2 Department of Experimental Medical Science, Lund University, SE-221 84, Lund, Sweden; 3 Chemistry Department, Princeton University, Princeton, New Jersey, 08544, United States of America; 4 SARomics Biostructures AB, Medicon Village, SE-223 81, Lund, Sweden; Russian Academy of Medical Sciences, RUSSIAN FEDERATION

## Abstract

The aminopropyltransferase spermidine synthase (SpdS) is a promising drug target in cancer and in protozoan diseases including malaria. *Plasmodium falciparum* SpdS (*Pf*SpdS) transfers the aminopropyl group of decarboxylated S-adenosylmethionine (dcAdoMet) to putrescine or to spermidine to form spermidine or spermine, respectively. In an effort to understand why efficient inhibitors of *Pf*SpdS have been elusive, the present study uses enzyme activity assays and isothermal titration calorimetry with verified or predicted inhibitors of *Pf*SpdS to analyze the relationship between binding affinity as assessed by K_D_ and inhibitory activity as assessed by IC_50_. The results show that some predicted inhibitors bind to the enzyme with high affinity but are poor inhibitors. Binding studies with *Pf*SpdS substrates and products strongly support an ordered sequential mechanism in which the aminopropyl donor (dcAdoMet) site must be occupied before the aminopropyl acceptor (putrescine) site can be occupied. Analysis of the results also shows that the ordered sequential mechanism adequately accounts for the complex relationship between IC_50_ and K_D_ and may explain the limited success of previous efforts at structure-based inhibitor design for *Pf*SpdS. Based on *Pf*SpdS active-site occupancy, we suggest a classification of ligands that can help to predict the K_D_−IC_50_ relations in future design of new inhibitors. The present findings may be relevant for other drug targets that follow an ordered sequential mechanism.

## Introduction

Malaria is one of the world’s deadliest diseases, affecting mainly economically weak countries in sub-Saharan Africa, where children under 5 years of age comprise 65% of the 473,000 yearly deaths [1]. Ninety percent of severe malaria cases are caused by the eukaryotic parasite *Plasmodium falciparum*. Recent reports of artemisinin-resistant *P*. *falciparum* strains in Southeast Asia emphasize the urgent need for new antimalarial drugs [2,3].

The polyamines putrescine, spermidine, and spermine play essential roles during cell division and proliferation [4–6]. Thus, the polyamine biosynthetic pathway has been a target for the design of drugs against cancer and various protozoan diseases over the last decades [5,7]. Inhibition of polyamine synthesis by the drug eflornithine is today the first-line treatment for West African sleeping sickness caused by the protozoan parasite *Trypanosoma brucei gambiense* [8]. Eflornithine is a specific inhibitor of the enzyme ornithine decarboxylase (ODC), which catalyzes the formation of putrescine from ornithine. Spermidine synthase (SpdS) and spermine synthase (SpmS) catalyze transfer of the aminopropyl group of decarboxylated S-adenosylmethionine (dcAdoMet) to putrescine, creating spermidine, or to spermidine, creating spermine, and producing 5′-methylthioadenosine (MTA) from dcAdoMet. Formation of dcAdoMet is catalyzed by S-adenosylmethionine decarboxylase. In *P*. *falciparum* the polyamine pathway is less complex than in higher eukaryotes, with several features that might be exploited for drug development [9,10]). Relevant to the present work is that *P*. *falciparum* lacks a specific SpmS and that its spermidine synthase (*Pf*SpdS) can also catalyze the formation of spermine from spermidine to a small extent [11]. Several inhibitors of mammalian SpdS that also inhibit *Pf*SpdS have been identified during the last decades. However, none of these has passed the preclinical stage of drug development for various reasons.

**Fig 1:**
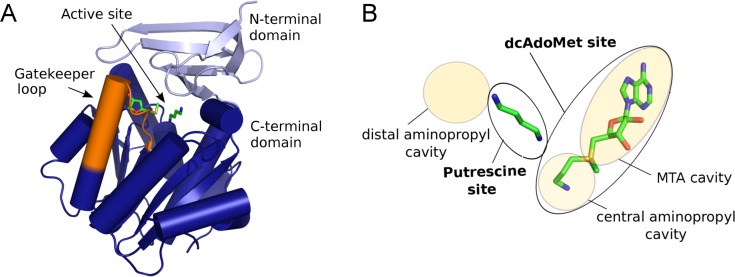
Overall structure and active site of *Pf*SpdS. (A) Monomer architecture. The N-terminal beta-sheet domain is light blue; the C-terminal Rossmann fold domain is dark blue. The active site is indicated in a cleft between the two domains, marked by a stick model of bound MTA and putrescine (green, based on PDB ID: 4BP1). The gatekeeper loop spanning the active site is shown in orange; when ligands are bound it adopts a loop-3_10_ helix-loop structure that is approximated in the representation shown. (B) Active site. Labeled yellow shaded or outlined oval shapes of different sizes represent the indicated parts of the active site referred to in the text. The larger dcAdoMet site (black-outlined oval at right) is conceptually divided into an MTA cavity (large upper shaded oval) and a central aminopropyl cavity (small lower shaded oval). The putrescine site (central black-outlined oval) is adjacent to a distal aminopropyl cavity (shaded oval at upper left). The substrates dcAdoMet and putrescine are represented as stick cartoons with green carbon atoms and other atoms in atomic colors (blue nitrogen, red oxygen, and yellow sulfur); these substrates do not occur together in any existing crystal structure because the enzyme reaction would occur. The cartoon is a composite based on separate structures with dcAdoMet and with MTA and putrescine.

Crystal structures of SpdS from *P*. *falciparum* and other eukaryotic and prokaryotic organisms have been solved in the last decade [12–16]. The SpdS fold (Fig 1A) consists of an N-terminal beta-sheet domain and a larger C-terminal Rossmann fold domain that belongs to the MTA methylase I fold class [12]. The active site is located in the cleft between the two domains [17] and comprises a larger binding site that accommodates dcAdoMet and a smaller binding site accommodating putrescine (Fig 1B). The putrescine site of *Pf*SpdS is flanked by an additional aminopropyl cavity extending beyond the distal nitrogen of putrescine, presumably accounting for the reported ability of *Pf*SpdS to convert spermidine to spermine [11]. The dcAdoMet site is subdivided into a small central cavity accommodating the donor aminopropyl group, and a larger cavity accommodating the MTA portion of the dcAdoMet substrate. In most SpdS enzymes the active site is spanned by a short polypeptide segment from the C-terminal domain (residues 196–208 in *Pf*SpdS) that forms several interactions with both the aminopropyl donor and acceptor substrates. This segment is referred to as the gatekeeper loop and it forms a loop-3_10_ helix-loop structure when ligands are bound in the dcAdoMet site, but is disordered in absence of ligands [12,18].

**Fig 2:**
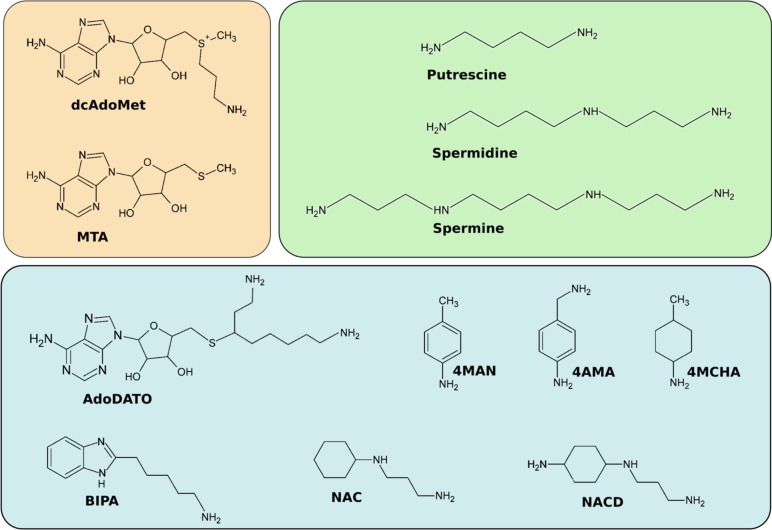
Chemical structures of *Pf*SpdS ligands. Upper left (beige), compounds that bind as substrate (dcAdoMet) and product (MTA) in the dcAdoMet site; upper right (green), natural substrates and products that bind in the putrescine site; lower (blue), inhibitors and other ligands discussed in this work, from left to right and top to bottom: S-adenosyl-3-thio-1,8-diaminooctane (AdoDATO); 4-methylaniline (4MAN); 4-aminomethylaniline (4AMA); cyclohexylamine; trans-4-methylcyclohexylamine (4MCHA); 5-(1H-benzimidazol-2-yl)pentan-1-amine (BIPA); *N*-(3-aminopropyl)-trans-cyclohexylamine (NAC); (1*R*,4*R*)-(*N*1-(3-aminopropyl)-trans-cyclohexane-1,4-diamine (NACD).

Crystal structures have also been solved of *Pf*SpdS in complex with various verified or predicted inhibitors [18–20]. The structures of compounds discussed in this work are shown in Fig 2, and their inhibitory properties are given in Table 1. AdoDATO (S-adenosyl-3-thio-1,8-diaminooctane, [21]) and 4MCHA (trans-4-methylcyclohexylamine) are among the most potent *Pf*SpdS inhibitors discovered yet [11,18,20]. AdoDATO is a multi-substrate analog that occupies both the dcAdoMet and putrescine substrate binding sites of *Pf*SpdS, whereas 4MCHA occupies only the putrescine site, and only in the presence of dcAdoMet [18). 4MCHA has been demonstrated to strongly inhibit *P*. *falciparum* cell growth *in vitro* at micromolar concentrations, confirming that *Pf*SpdS is a potential drug target in the parasite [11]. Two other aromatic amines, 4MAN (4-methylaniline) and 4AMA (4-aminomethylaniline) were also shown to bind to the putrescine site of *Pf*SpdS in presence of dcAdoMet and MTA, respectively [20].

**Table 1:** Properties of *Pf*SpdS inhibitors.

Compound	IC_50_	K_i_	Concentration in assay		Inhibition type	Reference
			[dcAdoMet]	[Putrescine]		
4AMA	> 1 mM	n.d.	100 μM	100 μM	n.d.	this work
4MAN	23 μM	8.2 μM	100 μM	100 μM	competitive; putrescine	this work
4MCHA	1.4 μM	0.2 μM	100 μM	200 μM	competitive; putrescine	[11]
AdoDATO	8.5 μM	3.4 μM*	100 μM	100 μM	mixed; putrescine	[18] and this work
BIPA	> 1mM	n.d.	100 μM	100 μM	n.d.	[22] and this work
MTA	159 ± 27 μM	n.d.	100 μM	200 μM	product inhibition	[11]
NAC	7.4 ± 2.4 μM	2.8 μM	350 μM	250 μM	mixed; putrescine	[19]
NACD	619 ± 144 μM	n.d.	350 μM	250 μM	n.d.	[19]

n.d., not determined due to weak binding

* K_i_ competitive

In a structure-based virtual screening study Jacobsson et al. [22] identified seven novel potential inhibitors of *Pf*SpdS among 2.6 million screened structures. Using NMR spectroscopy, one of these compounds, BIPA (5-(1H-benzimidazol-2-yl)pentan-1-amine), was reported to bind strongly to the enzyme [22]. Crystallization of *Pf*SpdS with BIPA [20] confirmed the binding orientation predicted in the *in silico* study [22]. However, neither BIPA nor any of the other six potential inhibitors showed any significant inhibitory effect (IC_50_ >> 100 μM) in *Pf*SpdS assays (L. Persson, unpublished results). In another virtual screening study Burger et al. [19] used dynamic pharmacophore models to identify novel *Pf*SpdS inhibitors, but again none of the selected compounds showed significant inhibition (IC_50_ >> 100 μM). In the same study rational design identified two inhibitors, NAC (4-N-(3-aminopropyl)-trans-cyclohexane-1-amine) and NACD ((1*R*,4*R*)-(*N*1-(3-aminopropyl)-trans-cyclohexane-1,4-diamine), with IC_50_ values of 7.4 μM and 619 μM, respectively. NAC had earlier been identified as a potent inhibitor of mammalian SpmS, but not of SpdS [23].

Thus, the ability of compounds to bind to *Pf*SpdS does not simply correlate with their apparent inhibitory activity. This observation, together with results from previous crystallographic studies, which indicate that *Pf*SpdS follows an ordered sequential substrate-binding mechanism [18,20], suggest that the limited success of *in silico* screening efforts to identify strong inhibitors of *Pf*SpdS is related to the enzyme’s reaction mechanism. As discussed previously by Cheng and Prusoff [24], in an ordered sequential mechanism the relation between IC_50_ and K_D_ depends on the concentration(s) of substrate(s) and on inhibition type (competitive with respect to the first or the second binding substrate, non-competitive, or uncompetitive), all factors which need to be taken into account in any inhibitor design efforts.

The present study combines enzyme assays with isothermal titration calorimetry (ITC) to evaluate the binding and inhibition of *Pf*SpdS by compounds with known or predicted inhibitory activity as well as the enzymatic mechanism. The ITC results presented here support a sequential ordered mechanism for *Pf*SpdS in which dcAdoMet binding is required before the polyamine substrate putrescine or alternative substrate spermidine is able to bind. Comparison of IC_50_ and K_D_ values for the selected set of compounds studied here confirms that binding and inhibition do not have a simple correlation. Based on the results, *Pf*SpdS ligands are classified according to which active-site cavities they occupy. It is proposed that this classification may be used to predict the expected correlations between IC_50_ and K_D_. The insights derived from the present analysis have implications for the design and interpretation of *in silico* and *in vitro* experiments aimed at identifying new inhibitors, not only for *Pf*SpdS, but also for other multi-substrate enzymes that follow a sequential order of binding events.

## Materials and Methods

### Materials

4AMA, MTA, putrescine and spermidine were purchased from Sigma-Aldrich (Stockholm, Sweden). BIPA and 4MAN were acquired from Princeton Biomolecular Research, Inc. (Princeton, NJ, USA). NAC and NACD was a kind gift from Dr. L-M Birkholtz and Dr. AI Louw (University of Pretoria, South Africa). dcAdoMet was generously supplied by Dr. A Shirahata and Dr. Y Ikeguchi (Josai University, Japan). AdoDATO was kindly supplied by Dr. AE Pegg and Dr. D Feith (Pennsylvania State University College of Medicine, Hershey, PA, USA).

### Protein expression and purification

Protein expression and purification was done as described previously [18,20]. For heterologous expression in *E*.*coli* (BL21(λDE3)-Rosetta, Oxford) the *Pf*SpdS construct with truncated N-terminus in p15-Tev-LIC vector was used. The cell extract after expression at 37°C was applied on Ni-affinity chromatography followed by gel filtration in running buffer (100 mM HEPES pH 7.5, 500 mM NaCl,) as described by Sprenger et al. [20]. Pure fractions after size exclusion chromatography were collected and concentrated to ~ 1 mg/ml using Amicon Ultra-15 centrifugal filter units (10,000 kDa).

### Spermidine synthase activity assay

A 39-residue-truncated *Pf*SpdS shortened from the N-terminus was used for all studies reported here. Its specific activity and Km for putrescine (482 nmol/mg/min and 36 μM, respectively; data not shown) are in good agreement with values (~500 nmol and 52 μM, respectively) reported earlier for a 29-residue-truncated *Pf*SpdS [11]. Spermidine synthase activity was determined by measuring the formation of spermidine from the substrates dcAdoMet and putrescine. The standard reaction mixture contained 0.1 mM dcAdoMet (> 95% purity as determined by HPLC), 0.1 mM putrescine, 1 mM DTT, 1 mM EDTA, 10 μg BSA, 0.2 μg *Pf*SpdS and 50 mM potassium phosphate buffer, pH 7.5, in a total volume of 100 μl. The enzyme assays were incubated for 30 min at 37°C and terminated by addition of 100 μl of 0.4 M HClO_4_. The amount of spermidine formed was determined by HPLC with *θ*-phthaldialdehyde as the reagent essentially as described by Seiler & Knödgen [25].

Determination of IC_50_ values was performed in standard assays containing increasing concentrations of inhibitors. For the determination of Km and Ki values of putrescine, 4MAN and AdoDATO, concentrations between 12.5–200 μM (putrescine), 20–100 μM (4MAN) and 5–25 μM (AdoDATO) were used with 100 μM dcAdoMet.

### Isothermal titration calorimetry

Protein samples of 1 mg/ml were dialyzed overnight at 4°C against buffer containing 100 mM HEPES, pH 7.5, 250 mM NaCl. Protein concentration was determined by UV absorption (A_280_ = 1 for 1.3 mg/mL) immediately before loading into the calorimeter cell. The dialysate was used for dilution of the ligand to concentrations of 0.3 to 1.0 mM from stocks prepared in DMSO at the highest concentrations allowed by their solubility limits (determined empirically). The protein at a concentration of 0.8–30 μM was loaded into the sample cell of a VP-ITC microcalorimeter (MicroCal, Inc.). All measurements were performed at 25°C with 25 injections of the ligand of 10 μl each delivered in 20 s with 240 s delays between injections. Titration of dialysis buffer or dialysis buffer containing 10% DMSO into protein resulted in undetectable dilution heat. Titrations of ligands into dialysis buffer were performed additionally and in case of non-negligible dilution heat the dilution profile(s) were subtracted point-by-point from the reaction profile. The dilution factor was taken into account for the binding curve fitting. Prior to titration of a second ligand into protein in presence of a first ligand, titration of dcAdoMet or MTA was carried out to saturating concentrations. For titration experiments with only dcAdoMet, MTA, AdoDATO, BIPA, spermidine and putrescine two independent measurements were performed, and for all other titration experiments only one measurement was performed. The results from independent measurements coincide, and values are shown for only one measurement. The ligand- and buffer-subtracted integrated binding data were fitted using a single-site binding model in MicroCal Origin software.

## Results

### Inhibition of *Pf*SpdS

The inhibitory properties of eight compounds whose binding to *Pf*SpdS is analyzed here are collected in Table 1. Five of the entries represent literature values of K_i_ and/or IC_50_ published previously. For the remaining three compounds, 4AMA, 4MAN, and BIPA, *Pf*SpdS activity was measured in this work with the typically used assay concentrations of 100 μM each of dcAdoMet and putrescine. Inhibitory activity of each potential inhibitor was initially evaluated at concentrations of 0.1 and 1 mM to identify those inhibiting strongly enough to warrant measurement of K_i_ and IC_50_ values. The results show that only 4MAN inhibits *Pf*SpdS at concentrations below 1 mM, having an IC_50_ value of 23 μM, similar to the IC_50_ value of 108 μM reported with mammalian SpdS [23]. 4AMA and BIPA show no or only minor inhibitory effects on *Pf*SpdS activity at concentrations of 0.1 or 1 mM (data not shown). The other six compounds identified by Jacobsson et al. [22] were also tested, and none of them inhibit *Pf*SpdS at a concentration of 0.1 mM (data not shown).

**Fig 3:**
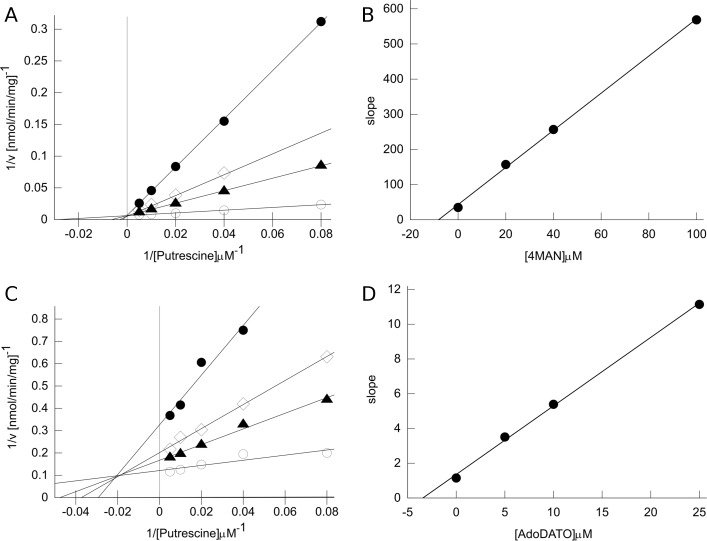
Inhibition of *Pf*SpdS activity. (A) Lineweaver-Burke plot for 4MAN. Reciprocal velocity *vs*. reciprocal putrescine concentration at 0.1 mM dcAdoMet in presence of 4MAN at final concentrations of 0 μM, open circles (○); 20 μM, filled triangles (▲); 40 μM, open diamonds (◊); or 100 μM, filled circles (●). Solid lines are linear regression fits for each 4MAN concentration. Each symbol represents the average of two technical replicates from one independent measurement (range = < 17%). (B) Secondary plot for 4MAN. The slope of each line in panel A is plotted *vs*. 4MAN concentration. The solid line is the linear regression fit, yielding the K_i_ value of 8.2 μM from the abscissa intercept. (C) Lineweaver-Burke plot for AdoDATO. Reciprocal velocity *vs*. reciprocal putrescine concentration at 0.1 mM dcAdoMet in presence of AdoDATO at final concentrations of 0 μM, open circles (○); 5 μM, filled triangles (▲); 10 μM, open diamonds (◊); or 25 μM, filled circles (●). Solid lines are linear regression fits for each AdoDATO concentration. Each symbol represents the average of two technical replicates from one independent measurement (range = < 9%). Two independent measurements were performed with reproducible results. (D) Secondary plot for AdoDATO. The slope of each line in panel C is plotted *vs*. AdoDATO concentration. The solid line is the linear regression fit, yielding the K_i_ competitive value of 3.4 μM from the abscissa intercept.

The inhibition of *Pf*SpdS by 4MAN was determined in this work to be competitive with putrescine as judged by Lineweaver-Burke plots (Fig 3). The plots show a common V_max_ but altered K_m_ at increasing inhibitor concentrations. Replotting the slopes *vs*. inhibitor concentration yields a K_i_ value of 8.2 μM from the negative x-axis intercept. Like 4MAN, 4MCHA has also been reported to be a competitive inhibitor with respect to putrescine, with an IC_50_ value of 1.4 μM and K_i_ value of 0.18 μM [11]. Competition with putrescine is consistent with the results of crystallographic analysis [18,20] showing that each of these compounds occupies the putrescine binding site of *Pf*SpdS. AdoDATO shows mixed-type inhibition with putrescine, with both V_max_ and K_m_ altered at increasing inhibitor concentrations (Fig 3). The intersection of the lines at a negative x-axis value and non-zero y-axis value indicates mixed inhibition. Replotting the slopes *vs*. inhibitor concentration yields a K_i_ competitive value of 3.4 μM from the negative x-axis intercept. The K_i_ uncompetitive value is estimated to be 12.2 μM (results not shown).

The rank order among the inhibitors of *Pf*SpdS in Table 1, from strongest to weakest inhibition according to the IC_50_ values, is 4MCHA, NAC, AdoDATO, 4MAN, MTA, NACD, 4AMA, and BIPA, with an overall range of IC_50_ values from ~ 1 μM to > 1 mM. The finding that 4AMA and BIPA are the weakest inhibitors in this group is the most unexpected, as BIPA was shown to be a strong binder by NMR [22] and both compounds were found to occupy the *Pf*SpdS active site in crystals [20].

### Isothermal titration calorimetry (ITC)

**Fig 4:**
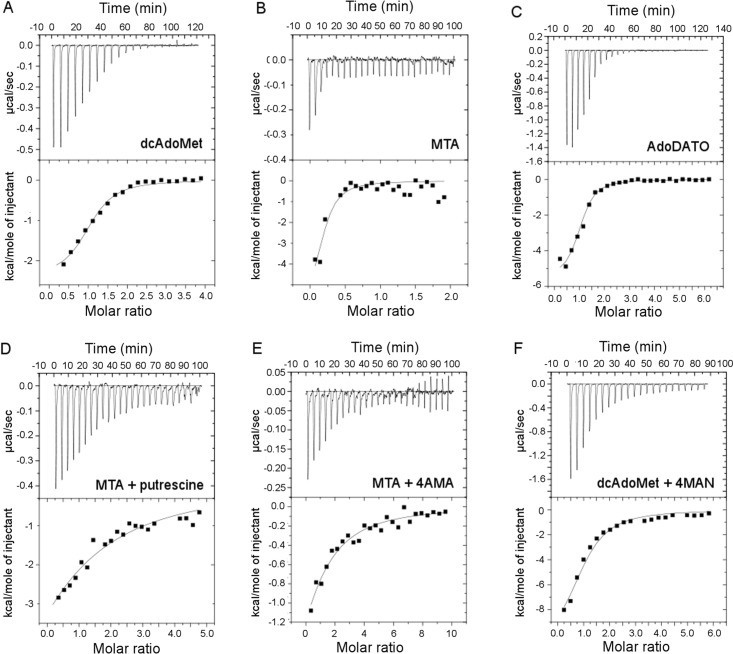
Ligand binding to *Pf*SpdS determined by ITC. *Pf*SpdS in the reaction cell was titrated with each indicated ligand in the syringe. Each upper panel shows the raw thermal data, and each lower panel shows the integrated binding heats (black squares) and the best nonlinear regression fit using a single-site binding model (solid line). The solid line in each lower panel is described by the thermodynamic parameters given in Table 2. Representative results are shown for selected ligands: (A) Titration of dcAdoMet into ligand-free *Pf*SpdS. (B) Titration of MTA into ligand-free *Pf*SpdS. (C) Titration of AdoDATO into ligand-free *Pf*SpdS. (D) Titration of putrescine into *Pf*SpdS saturated with MTA. (E) Titration of 4AMA into *Pf*SpdS saturated with MTA. (F) Titration of 4MAN into *Pf*SpdS saturated with dcAdoMet.

To additionally investigate the interdependence between pairs of compounds in binding to the enzyme, ITC was used to determine the binding affinity of substrates and predicted inhibitors to free *Pf*SpdS, or to *Pf*SpdS saturated with MTA or dcAdoMet. Control titrations of ligand(s) into buffer in most cases yielded heats of dilution that are negligibly small compared with the binding heats, as indicated by the magnitudes of the late peaks in the representative raw ITC data shown in Fig 4, or they were subtracted point-by-point from the reaction heats. All the integrated ITC binding data could be fitted adequately using a single-site model considering protein concentration as the monomer, with the exception of NACD in presence of MTA as discussed below. The parameters ΔH, ΔS and K_D_ recovered from the fits are listed in Table 2; the reported errors in the parameters are errors of the fits, and were not propagated to ΔS. The solution conditions for which the reported parameter values apply are 100 mM HEPES pH 7.5, 250 mM NaCl, 25°C. The steepness of most titrations (representative titration curves shown in Fig 4) indicates that most ligands show very strong binding under the conditions used, although some ligands yield detectable binding only in presence of other ligands. Compounds with K_D_ values weaker than ~ 300 μM, the detection limit under the conditions used, are reported as not binding.

**Table 2:** Thermodynamic parameters for ligand binding to *Pf*SpdS.

Ligand 1	Ligand 2^c^	ΔH, kcal/mol	ΔS, cal/mol/deg	K_D_, [μM]
dcAdoMet	-	-2.4 ± 0.2	16.7	3.7 ± 0.7
MTA	-	-2.7 ± 0.3	18.6	0.9 ± 0.5
AdoDATO	-	-5.5 ± 0.2	6.9	2.8 ± 0.5
Putrescine	-	-	-	>300
Spermidine	-	-	-	>300
4MAN	-	-	-	>300
4AMA	-	-	-	>300
NACD^a^	-	51.8 ± 2.5	196	8.4 ± 0.7
NACD^b^	-	17.6 ± 9.0	82.8	6.1 ± 1.6
NAC	-	-	-	>300
BIPA	-	1.5 ± 0.3	27.1	15.2 ± 8.2
dcAdoMet	4MAN	-11.4 ± 0.5	-15.0	7.7 ± 1.2
dcAdoMet	4AMA	-	-	>300
dcAdoMet	NAC	-1.0 ± 0.1	19.9	1.2 ± 0.8
MTA	putrescine	-12.4 ± 0.9	-22.4	59.5 ± 8.2
MTA	spermidine	-6.1 ± 0.3	6.74	1.2 ± 0.3
MTA	4MAN	-	-	>300
MTA	NAC	-	-	>300
MTA	4AMA	-3.5 ± 0.2	7.84	45.3 ± 5.1
MTA	NACD	-0.6 ± 0.1	28.4	0.3 ± 0.3

The errors for ΔH and KD are errors of the fit

^a^When two ligands were used, the thermodynamic parameters represent the binding of ligand 2 in the presence of saturating concentrations of ligand 1.

^b^Fit was done setting N = 1

^c^Fit was done letting N float resulting in N = 2.74

#### Titration into free enzyme

Titrations of the substrate dcAdoMet or the product MTA into *Pf*SpdS show strong, exothermic binding with affinities (K_D_ values) of ~ 4 and ~ 1 μM, respectively. The binding affinities for MTA and dcAdoMet are comparable to those reported for binding of decarboxylated S-adenosylhomocysteine to human SpdS (K_D_ = 1.1 μM at 20 mM sodium phosphate pH 7.5, 50 mM NaCl, 25°C; [26]). The binding affinities for AdoDATO and NACD are similar to those of dcAdoMet or MTA, with K_D_ values of ~ 3 μM and K_D_ ~ 8 μM, respectively, and with an endothermic binding heat for NACD. However, the number of NACD molecules binding to the protein was not well-constrained by the binding data despite several independent repetitions of the titration; values ranging from one to five NACD bound per monomer fit almost equally well using the one-site model, and a two-site model also gave comparable fit. BIPA binds with slightly lower affinity (K_D_ ~ 15 μM), and like NACD the binding heat is endothermic. No specific thermodynamic or structural significance can be attached to the endothermic or exothermic nature of a ΔH value; the measured change in enthalpy of a system upon binding reflects a typically very small difference between the much larger internal energies of the free and bound states, and may be dominated by changes in the system (e.g., solvation, counter ion binding) that are thermodynamically complex and may be structurally ambiguous. Putrescine, spermidine, 4MAN, 4AMA, and NAC have no measurable affinity for free *Pf*SpdS under the conditions used.

#### Titration into dcAdoMet-saturated enzyme

4MAN and NAC bind with strong affinity (K_D_ ~ 8 and ~ 1 μM, respectively) to the enzyme in the presence of dcAdoMet, but neither compound binds detectably to *Pf*SpdS alone or in the presence of MTA. For 4MAN this finding is in agreement with the result from X-ray structural analysis showing that electron density for the compound is observed when crystallized in presence of dcAdoMet but not in presence of MTA [20]. No binding for 4AMA could be observed (K_D_ > 300 μM), which is in agreement with the absence of electron density for 4AMA when crystallized with *Pf*SpdS in presence of dcAdoMet [20]. Binding of putrescine and spermidine to *Pf*SpdS in the presence of dcAdoMet was not examined because the catalytic reaction is expected to occur, as it does also with NACD (L. Persson and J. Sprenger, unpublished results).

#### Titration into MTA-saturated enzyme

ITC shows that prior binding of the tight-binding reaction product MTA to the enzyme at saturating concentration greatly increases the binding affinity of putrescine, spermidine, or 4AMA, with the product spermidine binding as strongly as the product MTA itself, K_D_ ~ 1 μM. Putrescine and 4AMA have K_D_ values of ~ 60 and ~ 45 μM, respectively, in presence of MTA. Binding is exothermic for all three ligands under these conditions. The binding of NACD, which is a strong, endothermic binder to the free enzyme, is stronger and exothermic in presence of MTA, K_D_ ~ 0.3 μM. The presence of MTA has no measurable effect on the affinity of NAC or 4MAN, both showing no detectable affinity to *Pf*SpdS under the conditions used. For 4MAN this result agrees with the finding that this compound is detected in the structure only when crystallized with *Pf*SpdS in presence of dcAdoMet [20].

## Discussion

Comparison of the results from the inhibition and ITC binding studies shows that the binding affinity of a compound does not directly reflect its inhibitory strength. Many compounds, among them, e.g., both NAC and 4MAN, do not show binding to the free enzyme (K_D_ > 300 μM), but both are relatively strong inhibitors. Both ligands bind strongly in presence of dcAdoMet. In presence of MTA 4AMA binds moderately strongly and NACD very strongly but both inhibit the enzyme poorly. NACD, AdoDATO, BIPA, and MTA each bind strongly to the free enzyme, but only AdoDATO is a strong inhibitor. These discrepancies indicate that for *Pf*SpdS binding and inhibition do not have a simple relationship. The correlation between K_D_ and IC_50_ can be understood in the context of the enzyme mechanism with reference to the work of Cheng and Prusoff [24].

### Enzyme mechanism

**Fig 5:**
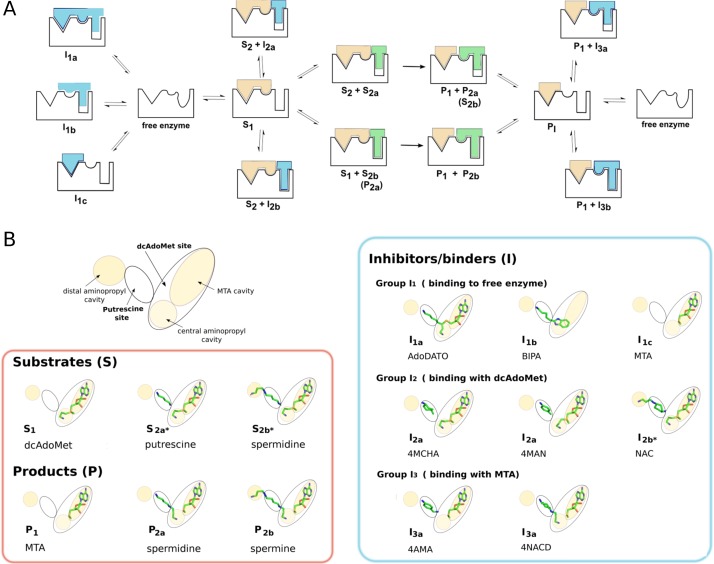
Ligand binding to *Pf*SpdS. (A) Pathways for substrates, products, and inhibitors. The enzyme is represented schematically by a black outline indicating its binding sites and their conformational changes upon ligand binding: left, MTA cavity (triangle; fully formed in the free enzyme, unchanged with bound ligands); middle, central aminopropyl cavity (distorted circle in the free enzyme; becomes circular with bound ligands); right, putrescine site plus distal aminopropyl cavity (distorted oval in the free enzyme; becomes rectangular with bound ligands, some of which do not fill the distal cavity completely). Coloring of schematically represented compounds follows the colored boxes in Fig 2: aminopropyl donor substrate and its product, beige; polyamine substrates and products, green; inhibitors, blue. The double arrows show binding equilibria between forms; the single arrows indicate enzymatic reaction. Letters and numbers under each shape correspond to the classes defined in Table 3 and depicted in panel B. (B) Classification of ligands. Upper left, the schematic representation of the active site introduced in Fig 1 and used here to identify ligand-binding locations. Lower left box, the indicated substrates and products are shown as stick cartoons placed in the appropriate active-site locations; right box, representation as in the left box showing the active-site positions of the compounds listed in Table 3. Numbers and letters below each entry correspond to classes defined in Table 3 and represented in panel A. Placements in the active-site locations are based on the respective crystal structures except for S_2a_* and S_2b_*, which are artificially positioned in the structure with dcAdoMet only based on structures P_2a_ and P_2b_ with MTA, and I_2b_*, which is artificially positioned in the structure with dcAdoMet based on structure I_3a_ of NACD with MTA.

An ordered sequential mechanism for the binding of substrates by *Pf*SpdS (Fig 5A and 5B) is strongly supported by the calorimetric titration results presented here, and consistent with previous observations from X-ray crystallographic analyses [18,20]. The results show that the aminopropyl donor substrate dcAdoMet (**S**_**1**_) has strong affinity for ligand-free *Pf*SpdS, while the aminopropyl acceptor substrates putrescine (**S**_**2a**_) and spermidine (**S**_**2b**_) have immeasurably weak affinity for the ligand-free enzyme under the conditions used here, but greatly increased affinity in the presence of the reaction product MTA. The observed sequential order of substrate binding is also in agreement with the kinetic analysis of Raina et al. [27] who suggested an ordered sequential or ordered random mechanism for mammalian SpdS. Release of products from the enzyme is also likely to be sequentially ordered, with the polyamine products spermidine (**P**_**2a**_) or spermine (**P**_**2b**_) released prior to MTA (**P**_**1**_). This inference is based on the fact that neither the product spermidine, nor the weak inhibitor 4AMA that may be considered a spermidine analog, binds to the ligand-free enzyme. Ligands that bind in the putrescine site have no detectable affinity when MTA is absent, implying they must dissociate simultaneously with or prior to MTA. Moreover, 4AMA does not bind in the presence of dcAdoMet, but like spermidine it binds in the presence of MTA (this work and [20]).

**Table 3:** Proposed classification of SpdS inhibitors.

Inhibitor group	Binds to	Example	Inhibitor type	IC_50_-K_D_ relation*	PDB entries
**I**_**1**_	free enzyme				
**I**_**1a**_	dcAdoMet and putrescine sites	**AdoDATO**	Competitive with dcAdoMet and polyamine substrate	Eq (2)	2I7C
**I**_**1b**_	aminopropyl and putrescine sites	**BIPA**	Competitive with dcAdoMet	Eq (1)	4CWA
**I**_**1c**_	dcAdoMet site	**MTA**	Competitive with dcAdoMet	Eq (1)	2HTE; 3RIE; 4BNO; 4BP1; 4CXM; 3B7P
**I**_**2**_	dcAdoMet-bound enzyme				
**I**_**2a**_	putrescine site	**4MAN;4MCHA**	Competitive with polyamine substrate	Eq (2)	4BP3; 2PT9
**I**_**2b**_	putrescine and distal aminopropyl sites	**NAC**	Competitive with polyamine substrate	Eq (2)	-
**I**_**3**_	MTA-bound enzyme				
**I**_**3a**_	putrescine and central aminopropyl sites	**4AMA; NACD**	Competitive with polyamine product	K_D_ << IC_50_	4BNO; 3RIE
**I**_**3b**_	putrescine and central and distal aminopropyl sites		Competitive with polyamine product	K_D_ << IC_50_	-

* Equation numbers in this Table are those used in the present work; the corresponding equation numbers of Cheng and Prusoff [24] are given in the main text.

The gatekeeper loop of *Pf*SpdS probably plays a key role in regulating the ordered sequential binding process. Comparison of crystal structures when only the dcAdoMet substrate is bound [PDB ID: 2PT6, [18]] or when the products MTA and putrescine are bound [PDB ID: 4BP1, [20]] shows identical conformations of the putrescine site residues, indicating that binding of dcAdoMet alone enforces an active conformation in the gatekeeper loop equivalent to the conformation it adopts in the product-bound state. In absence of ligands electron density for the gatekeeper loop residues 196–208 is either missing or shows an altered conformation compared to structures with ligands bound in the dcAdoMet site [18,20]. These results imply that the putrescine substrate site is formed by acquisition of structure in the gatekeeper loop when dcAdoMet is bound.

### Relation between IC50 and KD values

As noted over 40 years ago by Cheng and Prusoff [24], the relation between IC_50_ and K_D_ values for inhibitory compounds is not a simple one. In particular, the quantitative relationship between these two measures depends on a number of factors including: the kinetic reaction mechanism, i.e., single or multi-substrate, sequential or random ordered; the nature of the inhibition, i.e., competitive, non-competitive, or un-competitive; and the assay conditions, i.e., substrate concentrations. Particularly relevant for the present analysis is the fact that the correlation between inhibition and binding in an ordered sequential mechanism is different for compounds competing with the first or second binding substrate, or with a product. Based on these considerations the results presented here identify three inhibitor types depending on whether binding occurs when the dcAdoMet site is free, occupied by the first substrate dcAdoMet, or occupied by the product MTA.

#### Free enzyme

Inhibitors typified by BIPA and MTA are strong binders but poor inhibitors. In an ordered sequential mechanism, the IC_50_ values depend on K_D_ values as follows:

$${IC}_{50}=\frac{\left[A\right]}{K_{D,A}}∙\frac{\left[B\right]}{K_{M,B}}∙K_{D}$$ (1)

where [A] is the concentration of the first substrate, here dcAdoMet with dissociation constant K_D,A_ (3.7 μM); [B] is the concentration of the second substrate, here putrescine with Michaelis-Menten constant K_M,B_ (35 μM); K_D_ is the binding affinity of the inhibitor. Eq (1) above corresponds to equation (21) of Cheng and Prusoff [24]. Typically, the enzyme assay uses concentrations of 100–200 μM of both substrates dcAdoMet and putrescine. Consequently, IC_50_ values for compounds competing with dcAdoMet are expected to be at least ~100–200 times higher than their measured K_D_ values, which could explain the high IC_50_ value of 160 μM for MTA even though it binds with a strong affinity of 0.9 μM to free *Pf*SpdS.

#### dcAdoMet-bound enzyme

Inhibitors including the known putrescine competitors 4MCHA and 4MAN are relatively strong inhibitors with IC_50_ values in the low μM range. Under assay conditions dcAdoMet is at saturating concentrations relative to its K_D_ value, indicating that the active site is fully occupied by this substrate. The ability of an inhibitor to compete with putrescine then depends only on putrescine concentration. The correlation between IC_50_ and K_D_ values for this type of inhibitor is described by the simple case of competitive inhibitors with an enzyme binding one substrate:

$${IC}_{50}=\frac{\left[B\right]}{K_{M,B}}∙K_{D}$$ (2)

Eq (2) above corresponds to equation (6) of Cheng and Prusoff [24]. Under *Pf*SpdS assay conditions the measured IC_50_ values for such inhibitors are expected to be ~3–7 times higher than the corresponding K_D_ values. Cheng and Prusoff [24] also use this equation to describe compounds that show a mixed-type inhibition with the second binding substrate in an ordered sequential mechanism. Therefore, the low IC_50_ value of AdoDATO compared to other compounds that bind the free enzyme may be explained by its mixed-type inhibition with putrescine. Using Eq (2) to predict the IC_50_ value for AdoDATO under the assay conditions described in Table 1 with the measured K_D_ of 3.7 μM results in a predicted IC_50_ of 10 μM, which is in good agreement with the measured IC_50_ of 8.5 μM. For 4MAN the IC_50_ value of 23 μM was measured with 100 μM each of dcAdoMet and putrescine in the assay, and its IC_50_ value is ~3 times higher than its K_D_ of 7.7 μM. Also for NAC, the IC_50_ value of 7.4 μM is ~ 6 times higher than the measured K_D_ of 1.2 μM with 350 μM dcAdoMet and 250 μM putrescine in the assay [19].

#### MTA-bound enzyme

The relation between binding and inhibition has not been previously described to our knowledge for inhibitors that compete with the products of an enzyme that has sequential dissociation. This is the case here for inhibitors that are polyamine product analogs. Their lack of inhibition may be traced to their lack of binding under the assay conditions. Compounds of this group, e.g., 4AMA, have no affinity for the free enzyme and cannot compete with dcAdoMet for binding to the free enzyme, but they bind in presence of MTA. These compounds thus cannot bind the enzyme until the reaction produces some MTA from dcAdoMet. At this point they would compete well with the products spermidine or spermine by binding the enzyme when MTA is also bound. However, the assay measures the reaction in its initial phase where MTA concentrations are close to zero.

### Ligand classification

*In silico* methods used for drug design aim to predict potential active-site binders that inhibit enzyme activity. As discussed above, however, for an ordered sequential mechanism the relationship between binding and inhibition is dependent on the type of inhibition, and differs for compounds competing with the first or the second substrate (or both) [18,20] or with a product. Furthermore, the active site of *Pf*SpdS comprises several sites and cavities (Fig 1B). The type of inhibition that can be expected for a given compound depends on which sites or cavities it occupies and whether any other substrate or product affects its binding. For the *Pf*SpdS inhibitors or predicted inhibitors studied in this work the results of ligand-binding studies with free, MTA-bound, or dcAdoMet-bound enzyme, as well as activity assays, crystal structures, and/or *in silico* predictions, were used to classify the compounds into groups and subgroups, leading to the relationships between binding and inhibition shown in Table 3. The groupings are based on compounds studied to date, but can accommodate future members of the identified groups.

**Inhibitor group I**_**1**_ contains compounds that bind to ligand-free *Pf*SpdS and compete with dcAdoMet for binding to the free enyzme. A further sub-division considers whether both the dcAdoMet and putrescine sites are occupied (**Subgroup I**_**1a**_), the ligand binds to the central aminopropyl cavity and the putrescine site (**Subgroup I**_**1b**_), or the dcAdoMet site only is occupied (**Subgroup I**_**1c**_). **Subgroup I**_**1a**_ contains ligands like AdoDATO that occupy both the dcAdoMet and putrescine sites according to the crystal structure [18]. **Subgroup I**_**1b**_ includes ligands that bind in the putrescine site and also occupy the central aminopropyl cavity, but not the MTA cavity, of the dcAdoMet site; BIPA is such a ligand according to the crystal structure [20]. **Subgroup I**_**1c**_ contains ligands like MTA that bind in the dcAdoMet site. Adenosine, which is described as a binder by Jacobsson et al. [22] and is predicted to occupy the adenosine portion of the dcAdoMet site, may also belong to this subgroup. Additionally, decarboxylated S-adenosylhomocysteine, which occupies the entire dcAdoMet site of human SpdS and is reported to inhibit *Pf*SpdS [26], also belongs to this subgroup. NACD shows binding to the free enzyme according to the presented ITC data and hence may belong also in group 1; however, its binding pose is known only in complex with MTA and not with the free enzyme. Therefore, NACD is discussed below under group I_3_.

**Inhibitor group I**_**2**_ contains ligands that bind in the presence of dcAdoMet and can be seen as polyamine substrate competitors. A further sub-division considers whether the distal aminopropyl cavity is free, mimicking the position of putrescine (**Subgroup I**_**2a**_), or is occupied by the ligand, mimicking the position of spermidine in presence of dcAdoMet (**Subgroup I**_**2b**_). **Subgroup I**_**2a**_ includes ligands that bind in the putrescine site only when dcAdoMet is bound and that can be considered as putrescine substrate analogs. Previously studied representatives of this group are the known putrescine competitors 4MCHA and 4MAN for which crystal structures in presence of dcAdoMet are available [18,20]. **Subgroup I**_**2b**_ includes compounds that occupy the distal aminopropyl cavity in addition to the putrescine site and thus can be considered spermidine substrate analogs. This subgroup may contain inhibitors that are specific for the *Pf*SpdS and do not inhibit human SpdS, which lacks the additional aminopropyl cavity. NAC is the only representative of this subgroup to date and it does inhibit *Pf*SpdS and human SpmS, but not human SpdS [23]. *In silico* prediction [19] suggests that in presence of dcAdoMet this compound occupies the putrescine site and distal aminopropyl cavity (Fig 5B). This predicted position is in agreement with the ITC results presented here that show NAC binding with high affinity in the presence of dcAdoMet but not MTA, nor to the free enzyme.

**Inhibitor group I**_**3**_ contains compounds that bind to *Pf*SpdS in the presence of MTA and can be seen as polyamine product analogs as they occupy the central aminopropyl cavity partially or fully. This group contains compounds like 4AMA and NACD that in presence of MTA bind in the putrescine and central aminopropyl cavity and can be considered spermidine product analogs. 4AMA binds relatively strongly in the presence of MTA but not to the free or dcAdoMet-bound enzyme, and is a poor inhibitor. NACD, which binds with very high affinity in presence of MTA, is a weak inhibitor similar to 4AMA. A further differentiation of **group I**_**3**_ into subgroups depends on the occupancy of the distal aminopropyl cavity, analogous to **subgroups I**_**2a**_ and **I**_**2b**_. Members of **subgroup I**_**3a**_ include spermidine product analogs like 4AMA and NACD, and **subgroup I**_**3b**_ members include spermine analogs occupying the central aminopropyl cavity, the putrescine site, and the distal aminopropyl cavity. **Subgroup I**_**3a**_ shares similarities with **subgroup I**_**1c**_, as these ligands occupy the central aminopropyl cavity and the putrescine site. **Subgroup I**_**1c**_, however, has measurable affinity for the free enzyme. Future compounds predicted to bind the central aminopropyl cavity and the putrescine site will fall under **subgroup I**_**1c**_ when they have measurable affinity to the free enzyme and into **subgroup I**_**3a**_ when they bind in presence of MTA.

The findings of this work may apply as well to other enzymes that follow an ordered sequence of binding and where the transferred group is large enough to cause steric interference. In such cases the product after transfer is expected to have reduced affinity in presence of the donor substrate due to steric clash. Many enzymes with sequential substrate binding transfer only electrons, and these arguments may not apply unless the change in redox state causes large structural change.

A systematic classification like that proposed here may have predictive value in virtual screening aimed at identifying inhibitors of enzymes that follow a sequential binding mechanism. The scoring functions used for ligand screening are designed primarily to detect potential binders [28], whereas the enzyme assays used to validate the screening results often test competition with substrates, not binding *per se*. Understanding the correlation between inhibition (IC_50_) and binding (K_D_) should improve the outcome of the search for potent inhibitors. Cheng and Prusoff [24] explained the complex relationship between binding and inhibition and how this is affected by a variety of parameters, including enzyme mechanism, type of inhibition, and concentrations of substrates. Enzyme assays used to evaluate predicted inhibitors may contain high substrate concentrations compared to cellular concentrations; depending on the inhibitory mechanism, some strong inhibitors under *in vivo* conditions may show weak or no inhibition in an *in vitro* assay. A more reliable selection of compounds for further investigation can be achieved not only by choosing inhibitors with the lowest IC_50_ values in the assay but also by taking into account their K_D_ values, which can be directly measured or estimated from their IC_50_ values. *In silico* drug design should therefore consider not only the enzyme mechanism but also the binding affinity and concentrations of substrates present in the assays used to identify inhibitors, as well as the sites or sub-sites occupied by the potential inhibitors in presence and absence of substrates. Thus the present results have relevance for the development of inhibitors of other enzymes with complex binding sites and mechanisms.
